# Porric acid E, a natural compound from Rhytidhysteron sp. BZM-9, suppresses colorectal cancer growth via an autophagy-dependent pathway

**DOI:** 10.7150/jca.77588

**Published:** 2022-10-31

**Authors:** Da Tang, Wei Zhang, Zhenxing Zou, Yikun Wang, Shichao Yan, Sha Zhang, Wenwu Cai, Daming Li, Qiuguo Li, Wenbo Li

**Affiliations:** 1 Department of Plastic and Aesthetic (Burn) Surgery, the Second Xiangya Hospital of Central South University, Changsha, China.; 2 Xiangya School of Pharmaceutical Sciences, Central South University, Changsha, China.; 3 Department of General Surgery, the Third Xiangya Hospital of Central South University, Changsha, China.; 4 Department of Pharmacy, the Second Xiangya Hospital of Central South University, Changsha, China.; 5 Hunan Institute for Drug Control, Changsha, China.; 6 Department of General Surgery, the Second Xiangya Hospital of Central South University, Changsha, China.; 7 Department of Laboratory Medicine, the Second Xiangya Hospital of Central South University, Changsha, China.; 8 Hunan Key Laboratory of Diagnostic and Therapeutic Drug Research for Chronic Diseases, Changsha, China.; 9 Department of Intervention, Hunan Chest Hospital, Changsha, China.

**Keywords:** Colorectal cancer, Endophytic fungi, Natural compound, Apoptosis, Autophagy

## Abstract

Colorectal cancer (CRC) is one of the major killer diseases worldwide, and more effective therapeutic compounds for CRC treatment are urgently needed. Although bioactive natural products derived from endophytic fungi have been extensively employed as antibiotics and anticancer agents, little is known about the effect of Rhytidhysteron sp. BZM-9 (an endophytic fungus)-derived compounds on CRC. Herein, a natural molecule porric acid E was isolated from Rhytidhysteron sp. BZM-9. Alamar Blue cell viability assay, Western blotting, transmission electron microscopy, flow cytometry analysis, and fluorescence image examination were employed to evaluate the antitumor effects of porric acid E on CRC cell lines. To establish the xenograft tumor model, nude mice received subcutaneous implants consisting of CRC cells on their flanks. Then the mice were treated with porric acid E or vehicle to assess the tumor-killing effects. The results revealed that porric acid E exhibited cytotoxicity by inhibiting proliferation and promoting apoptosis in CRC cells *in vitro*. Additionally, compared with fluorouracil (5-FU), porric acid E exhibited a more potent inhibitory effect on CRC HT29 cells. Importantly, extensive autophagy induced by porric acid E was detected in CRC cells, whereas inhibition of autophagy could significantly ameliorate porric acid E-mediated cytotoxic effect on CRC cells. Moreover, porric acid E treatment could markedly suppress subcutaneous HT29 xenograft tumor growth *in vivo*. Bioinformatics prediction indicated that Beclin-1 might be the potential target of porric acid E. These findings might afford a useful and important method for the treatment of CRC through fungal endophyte-derived natural compounds.

## Introduction

Colorectal cancer (CRC) is considered to be the third most common cancer, and the fourth leading cause of cancer-related death worldwide 1, 2. Despite the tremendous progress made in the treatment of CRC, the survival rate of patients with unresectable lesions is as low as 13.1% 3. Surgical resection is the main therapeutic approach for patients with early-stage CRC. However, because of drug resistance or dose-limiting toxicity, only limited success was achieved in the treatment of patients with advanced CRC 4. Thus, there is an urgent need to develop novel therapeutic agents with fewer side effects and improved antitumor efficiency for CRC treatment.

Recently, natural compounds have received special attention in cancer therapeutics because of their potential ability to interfere with the carcinogenic processes by targeting several molecular signaling pathways or altering the tumor cell behaviors 5. Natural anticancer compounds with significant efficacy extracted from plants, microorganisms, and marine organisms have been used in clinical practice 6. Many natural products have been shown to suppress tumor growth by inducing cell cycle arrest or apoptosis 7, 8. Endophytic fungi constitute an enormous and comparatively untapped source of biodiversity that is considered a wellspring of effective novel natural products with potential medical applications 9. Since Taxol was first discovered in the endophytic fungus Taxomyces andreanae, endophytic fungi have drawn tremendous attention from researchers 10. Over the past two decades, many anticancer metabolites, including camptothecin, vinblastine, vincristine and their derivatives isolated from endophytic fungi are currently being used clinically for the treatment of various cancers 11. Our previous studies showed that four new chlorinated cyclopentene derivatives extracted from Rhytidhysteron sp. BZM-9 (an endophytic fungus) could moderately inhibit the cell viability of human CRC cell lines 12. To further obtain the bioactive part of Rhytidhysteron sp. BZM-9, we isolated a monomer compound porric acid E from this endophytic fungus and evaluated its antitumor effects on CRC cells.

Autophagy is a highly conserved cellular process that degrades and renews misfolded or dysfunctional proteins and aging/damaged organelles to maintain cellular homeostasis through the lysosomal pathway and has been shown to exert a variety of key roles related to cell survival, development and differentiation 13. Evidence has accumulated that autophagy is involved in tumor progression and responses to anticancer therapies 14. Some studies have suggested that the inhibitory effect of natural compounds is not only reflected in the administration of apoptosis but also prevents the progression of the disease by modulating the autophagy process, which indicates that natural products could directly or indirectly regulate the target proteins on the autophagic signal pathways 15. This might be a primary mechanism of some natural products in the treatment and prevention of diseases.

In this study, we investigated the inhibitory effect of an endophyte-derived compound porric acid E on CRC cells both *in vitro* and *in vivo*. Furthermore, we hypothesize that the autophagy-dependent pathway is indispensable for the anticancer activity of porric acid E.

## Materials and Methods

### General experimental procedures

NMR spectra were collected with a Bruker AV-600 MHz spectrometer (Bruker, Karlsruhe, Germany) using tetramethylsilane (TMS) as an internal reference and CD3OD as solvent. HRESIMS spectrum was recorded on an Agilent 6500 series Q-TOF mass spectrometer (Agilent, Singapore) instrument equipped with an electrospray ionization (ESI) probe operating in positive-ion mode. Silica gel (200-300 or 60-100 mesh, Qingdao Marine Chemical, Ltd., China) and RP-18 gel (40-75 mm, Fuji, Kasugai, Japan) were used for column chromatography (CC). Preparative HPLC was performed on an Agilent 1100 system with a YMC-peak ODS-A column (5 mm, 250 × 10 mm). All other chemicals used in this study were of analytical grade.

### Fungal material

The fermentation, extraction, and isolation of the fungal strain Rhytidhysteron sp. BZM-9 was performed as described previously 12.

### Extraction and isolation of porric acid E

The EtOAc extract (50 g) of the extract was subjected to silica gel CC eluting with a PE-EtOAc-MeOH gradient (v/v/v, 100:0:0 - 0:0:100) to produce ten fractions (Fr. 1 to Fr. 10) on the basis of TLC analysis. Fr. 6 was further purified by silica gel CC and eluted with CH_2_C_l2_-MeOH (v/v, 100:0 - 0:100) to afford eight subfractions (Fr. 6-1 to Fr. 6-8). Fr. 6-6 was fractionated by reversed-phase silica gel (RP-18), using a MeOH-H_2_O gradient (v/v, 20:80 - 100:0), further yielding seven subfractions (Fr. 6-6-1 to Fr. 6-6-7). Fr. 6-6-5 separated by reversed-phase semi-preparative HPLC using ACN-H_2_O (0-40 min, 30-40%) to yield porric acid E.

### Reagents

Western blot antibodies: β-actin, P62, Beclin-1, LC3, AMPK, p-AMPK, mTOR and p-mTOR antibodies were purchased from Cell Signaling Technology. Chloroquine (CQ) was obtained from Sigma-Aldrich. Apoptosis detection kit was purchased from Miltenyi. Alamar Blue cell ability assay kit was obtained from Promega. Flurouracil (5-FU) was purchased from Sigma Chemical Co. (St. Louis, MO, USA).

### Cell culture and treatments

NCM460 and the CRC cell lines HT29, SW620, CX-1 and LOVO were obtained from the Cell Resource Center of Shanghai Institutes for Biological Sciences. HepG2, 5-8F and MDA-MB-231 were obtained from School of Basic Medical Science, Central South University. These cell lines were authenticated by short tandem repeat (STR) profiling. Before the experiment, all cells were not contaminated with mycoplasma. For the *in vitro* study, all of these cell lines were seeded in culture dishes or 96-well plates and allowed to grow overnight, then porric acid E with or without the CQ was added into the cell media at various concentrations and continued to be incubated for 48 h.

### Cell proliferation/viability assay

To assess the effects of natural products on cell proliferation, CRC cell lines were seeded in 96-well plates at a density of 1 × 10^5^ cells/ml in a volume of 100 μl/well. 24 h later, the cells were treated with porric acid E in the presence or absence of CQ or Z-VAD-FMK and cultured for another 48 h. Cell proliferation/viability was examined using an Alamar Blue cell viability assay kit as previously described by our group 29. Briefly, 20 μL of Alamar Blue assay reagent was added to each well and incubate for 2 h. The fluorescent signaling was directly measured using a microplate reader at 530/590 nm. The assay is based on the capability of living cells to convert a redox dye (resazurin) into a fluorescent end product (resorufin), whereas nonviable cells fail to produce a fluorescent signal as a result of the loss of metabolic capacity. Finally, IC_50_ (half maximal inhibitory concentration) values were calculated using GraphPad Prism software.

### Cell apoptosis analysis

Cells were plated into 35 mm dishes at a density of 2 × 10^5^ cells/mL and treated with different concentrations of porric acid E for 48 h. Then, the cell death was evaluated using the Annexin V-FITC/PI apoptosis detection kit (Miltenyi) with a BD FACS Canto II flow cytometer (Franklin Lakes, NJ, USA) following the manufacturer's instructions.

### Western blot analysis

Cultured cells were lysed in lysis buffer with protease inhibitors and centrifuged at 16,000 g for 10 minutes, and the supernatant (whole cell lysates) was collected. Then the concentrations of these supernatants were determined with the BCA protein quantification method. All protein samples were denatured by boiling. The same amount of total protein for each sample was then separated by 8%, 10%, or 12% sodium dodecyl sulfate-polyacrylamide gel electrophoresis (SDS-PAGE). Next, samples were then transferred onto PVDF membranes at 250 mA for 2 h and transferred onto PVDF membranes. After that, blocked these membranes in 5% milk for 1 h and incubated the respective primary antibodies overnight. Membranes were washed three times in TBST for 15 minutes and then incubated with HRP-conjugated secondary antibody. Protein expression levels were analyzed using the ECL detection system.

### Organ damage assessment

Serum alanine aminotransferase (ALT) and blood urea nitrogen (BUN) levels were evaluated using the Opera Clinical Chemistry System and were documented as international units per liter and milligrams per deciliter, respectively.

### Transmission electron microscopy

HT29 cells were seeded into 60 mm dishes at a density of 5 × 10^5^ cells/mL and treated with or without porric acid E. 48 h later, the cells were collected, fixed with 2.5% glutaraldehyde, and then fixed in 1% osmium tetroxide (OsO_4_) at 4 °C for 1-2 h. After that, these cell samples were dehydrated in a graded series of acetone (50%, 70%, 90%, and 100%), and embedded in Epon-Araldite resin. Then, ultra-thin sections (50-100 nm) were treated with 3% uranyl acetate and lead nitrate. Morphological changes focused on the observation of autophagosomes and autolysosomes. Cell images were acquired by using an HT7700 transmission electron microscope (Hitachi, Tokyo, Japan).

### Immunohistochemistry (IHC) analysis

The tumor tissues were cut and fixed in 4% paraformaldehyde and then embedded in paraffin. Each sample was sliced into the 5-μm-thick section. All sections were then incubated with primary antibodies (P62 and LC3, CST) for 18 h at 4 °C. Then, the sections were incubated with horseradish peroxidase-conjugated goat anti-rabbit secondary antibody for another 1 h, and the diaminobenzidine (DAB)/H_2_O_2_ system was used for visualization. Finally, the images were captured with a LEICA DMi8 inverted fluorescence microscope.

### Animal model and treatments

BALB/c mice (female, 5-6 weeks old) were provided by Hunan SJA Laboratory Animals Co. Ltd. The *in vivo* studies were conducted following institutional guidelines of the Ethics Committee of Central South University (Changsha, China). To establish the xenograft tumor model, nude mice received subcutaneous implants consisting of 5×10^6^ HT29 cells on their flanks. 10 days later, when the tumor volumes reached up to 100 mm^3^, these mice were randomly divided into two groups. Then, the mice were treated with porric acid E every three days via tail vein injection at a dose of 3 mg/kg in 200 μL of saline to evaluate the tumor-killing effects. After that, tumor growth was recorded for three weeks by measuring the major (a) and minor (b) axes of these tumors using a vernier caliper weekly.

### In-silico prediction of the interaction between porric acid E and core target(s)

All tested protein-protein interactions were analyzed using the STRING database to screen the core target(s) of porric acid E. Moreover, the interaction between porric acid E and core target(s) was predicted by molecular docking. The protein crystal structure of humanized core target (4ddp) was obtained from PDB database, and the molecular structure of porric acid E was drawn by Chem 3D 2020 software. The molecular docking between porric acid E and core target was analyzed by the SwissDock online platform.

### Statistical analysis

All quantitative data were shown as mean ± standard error of the mean (SEM) from at least three independent experiments. Data analysis was achieved using GraphPad Prism software. Data comparisons between two experimental groups were analyzed by 2-tailed Student t test. One-way ANOVA analysis was performed for multiple group comparisons. *P* < 0.05 was considered statistically significant.

## Results

### Structural characterization of porric acid E

**Figure 1:**
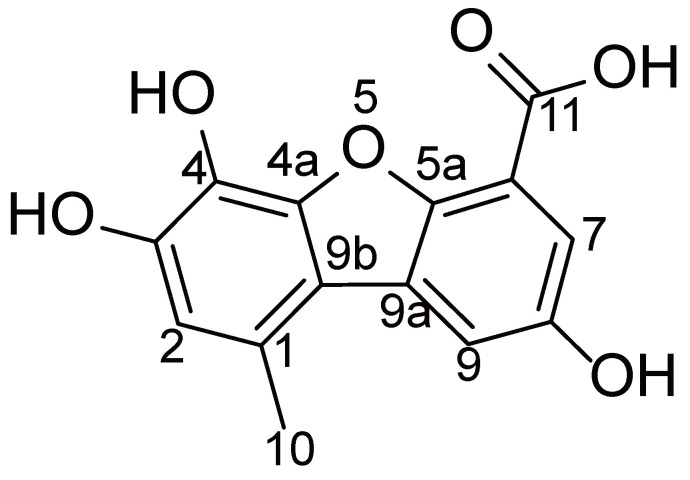
Structure of porric acid E.

Porric acid E was obtained as brown powder. HRESIMS and NMR spectra of porric acid E are shown in supplementary Fig. S1-S3. Its molecular formula was determined as C_14_H_10_O_6_ according to the HRESIMS peak at m/z 275.0555 [M + H] + (calculated for C_14_H_10_O_6_, 275.0556). The 1D NMR data (Table 1) of the compound was identical to porric acid E (Fig. 1), which was isolated from the EtOAc extract of the marine sponge-derived fungus Didymellaceae sp. SCSIO F46, and identified by comparing its NMR data with those previously reported in the literature 16. However, only its displayed COX-2 inhibitory activity has been reported so far.

### Porric acid E presents potent cytotoxicity on CRC cells

**Figure 2:**
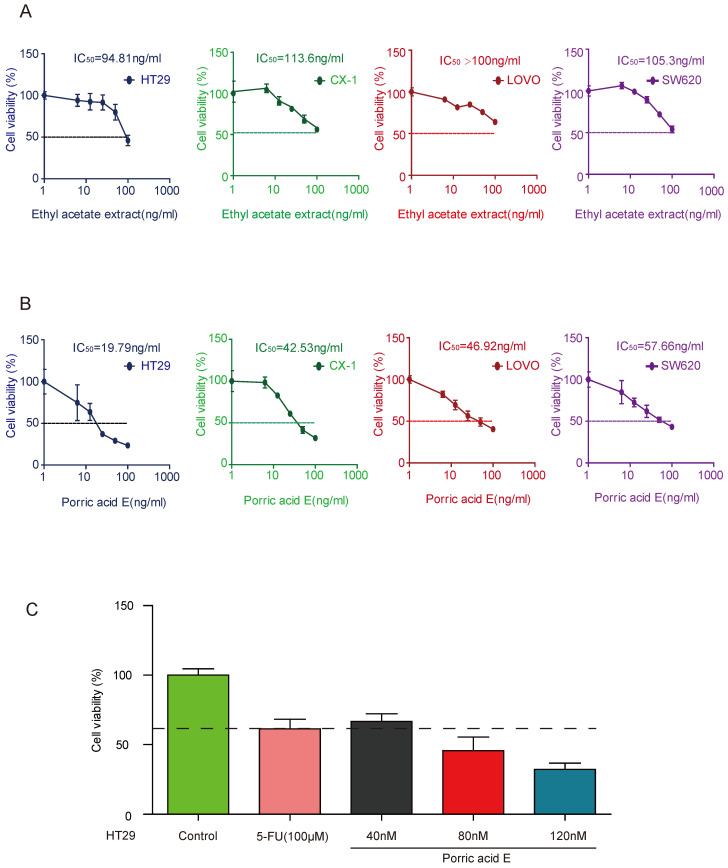
** Ethyl acetate extract and porric acid E exhibited a strong cytotoxic effect on CRC cells. (A)** HT29, CX-1, LOVO and SW620 cells were treated with various concentrations of ethyl acetate extract for 48 h and subjected to Alamar Blue cell viability assay. **(B)** CX-1, LOVO, HT29 and SW620 cells were exposed to different concentrations of porric acid E, and cell viability was detected by an Alamar Blue assay. **(C)** HT29 cells were treated with 5-FU or different doses of porric acid E, and Alamar Blue was used for the evaluation of cell viability.

In this study, we used four CRC cell lines (CX-1, HT29, SW620, and LOVO) as our *in vitro* model. To explore the effects of natural products on the viability of CRC cells, we first examined the effect of ethyl acetate extract derived from the strain Rhytidhysteron sp. BZM-9 on the proliferation of CRC cell lines. The results indicated that ethyl acetate extract treatment could inhibit the proliferation of these four cell lines as determined by Alamar blue cell viability assay (Fig. 2A). We further investigated the inhibitory effect of porric acid E, a monomer derivative isolated from ethyl acetate extract, on CRC cells. The findings also showed that porric acid E suppressed the proliferation of these cells in a dose-dependent manner (Fig. 2B). The optimal effect of porric acid E was observed on HT29 cells (IC_50_: 19.79 ng/ml=72nM). Hence, HT29 cell lines were chosen for our subsequent experiments. Fluorouracil (5-FU) is one of the most commonly used chemotherapeutic agents in CRC treatment 17. We further compared the inhibitory effect of porric acid E and 5-FU on HT29 cells by evaluating the cell viability. The data revealed that porric acid E at the low-dose range (nM) was even more effective than a high dose of 5-FU (μM) in inhibiting the viability of HT29 (Fig. 2C). Additionally, we also found that porric acid E could inhibit the proliferation of other cancer cells, but had little influence on the cell viability of normal human colon mucosal epithelial cell line (NCM460) (Supplementary Fig. S4). These results demonstrated that porric acid E is a multifunctional antitumor agent with strong cytotoxic effect on CRC cells.

### Porric acid E treatment induces autophagy in CRC cells

**Figure 3:**
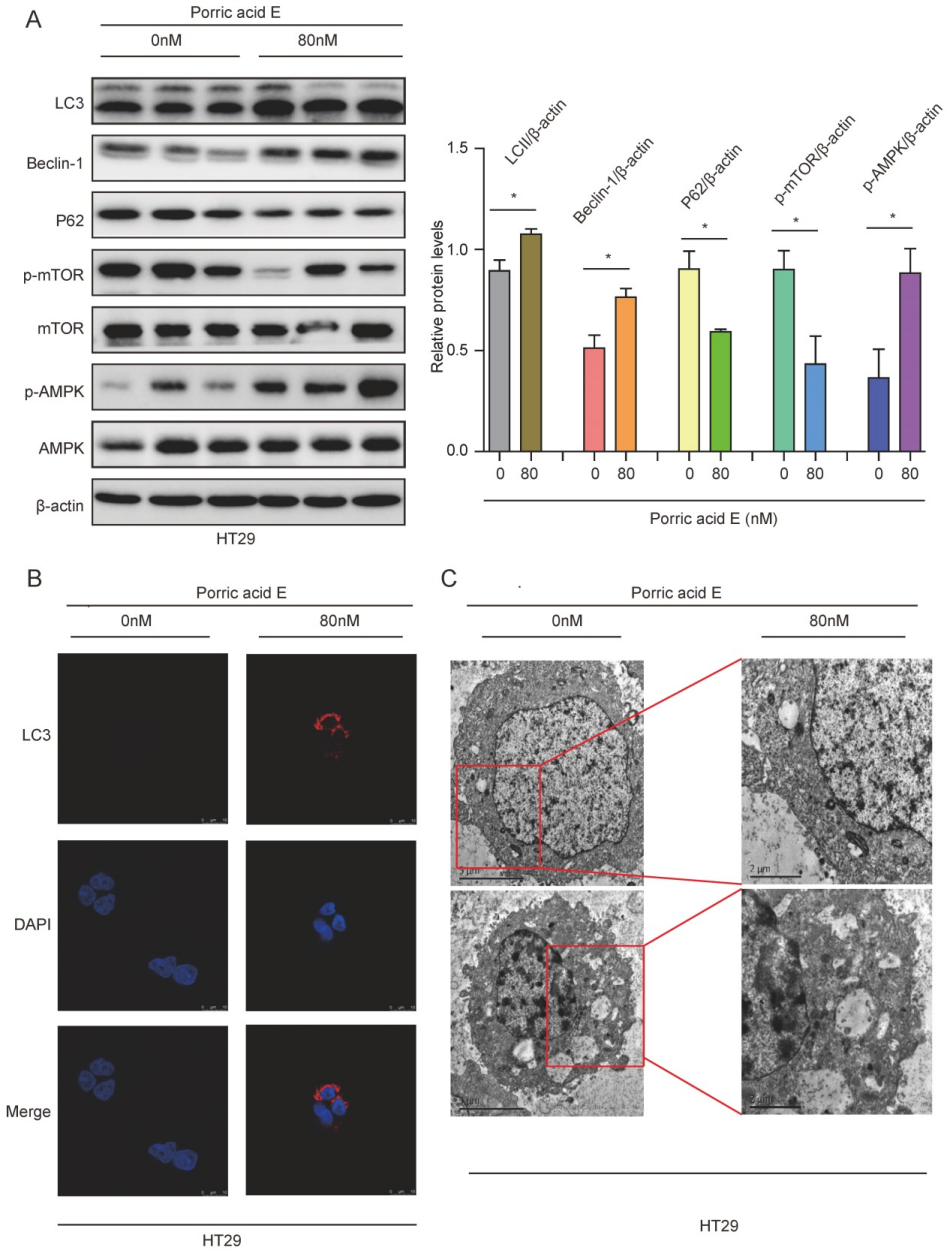
** Porric acid E induced autophagy in HT29 cells. (A)** The whole cell lysates of HT29 cells were harvested at 48 h after porric acid E treatment (80 nM). The levels of LC3, p62, Beclin-1, mTOR, p-mTOR, AMPK and p-AMPK were evaluated by western blot analysis. **(B)** 48 h post-porric acid E treatment, LC3 puncta were observed by immunofluorescence using a laser scanning confocal microscope. **(C)** Representative TEM images of HT29 cells treated with a vehicle or porric acid E treatment were collected 48 h after treatment. Data are presented as means± SEM. *P < 0.05.

Numerous investigations revealed that autophagy, which is commonly activated in response to radiotherapy or chemotherapy, may potentiate killing effects on different cancer types 18-20. To elucidate whether autophagy was involved in porric acid E mediated cytotoxicity in CRC cells, the autophagy-related proteins were analyzed by Western blot analysis. The increased protein levels of LC3B-II and Beclin-1 and decreased accumulation of p62 were observed in HT29 cells, indicating that porric acid E could induce autophagy in CRC cells (Fig. 3A). In addition, we found that the porric acid E-treated group expressed a significantly higher level of p-AMPK and a lower level of p-mTOR compared with the control group (Fig. 3A). Immunofluorescence assays also showed that porric acid E induced an increased number of LC3B puncta in HT29 cells (Fig. 3B). Furthermore, the levels of autophagy were examined by the formation of autophagosomes, as visualized using transmission electron microscopy (TEM). Normal morphology and no autophagosomes were detected in vehicle-treated HT29 cells, whereas a large number of autophagosomes were observed in porric acid E-treated cells (Fig. 3C). Taken together, these findings revealed that autophagy is involved in porric acid E induced cytotoxicity.

### Porric acid E-induced autophagy promotes cell death in CRC cells

**Figure 4:**
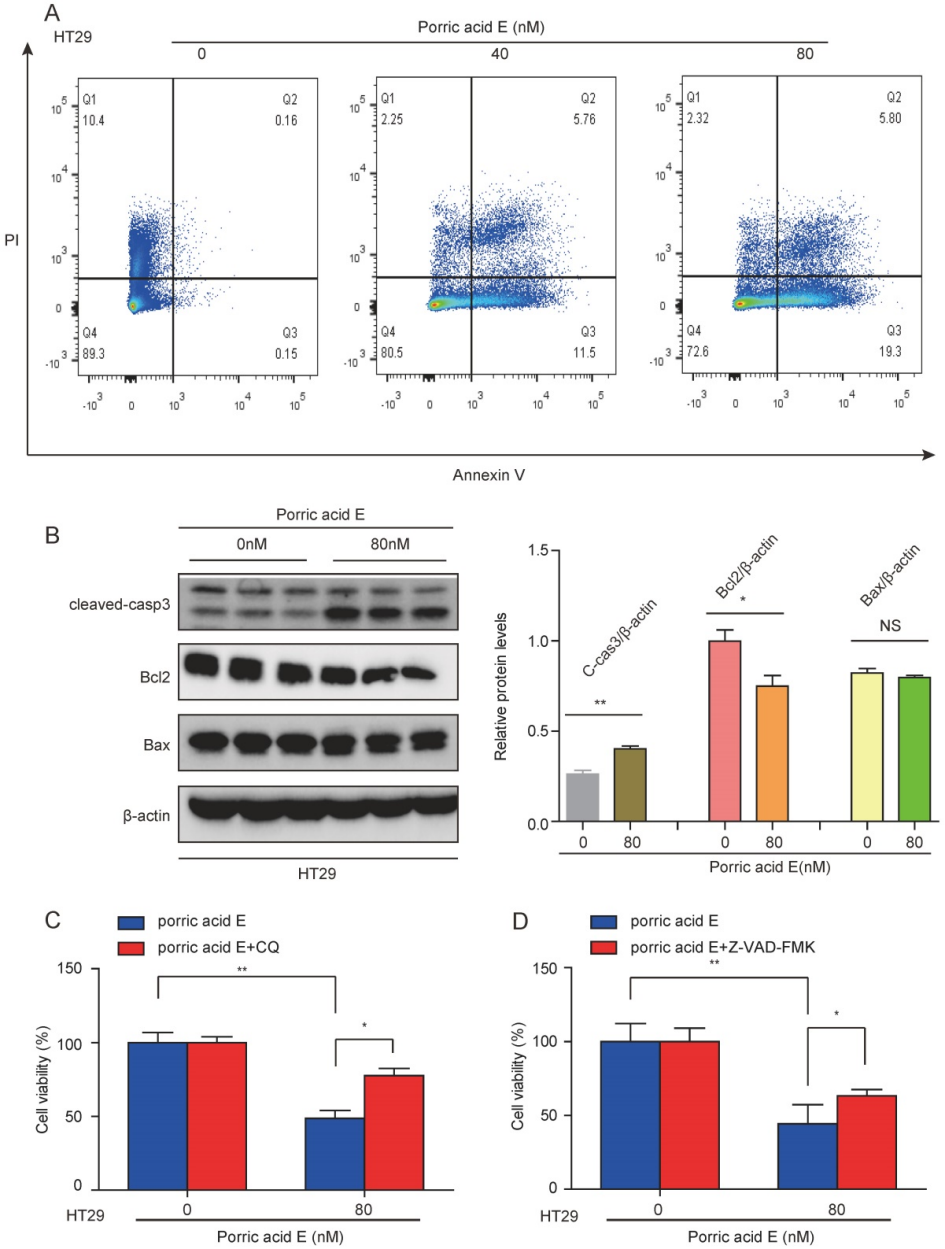
** Autophagic cell death was implicated in porric acid E-induced inhibitory effects in HT29 cells. (A)** After 48 h of treatment, flow cytometry was performed to assess cell apoptosis with Annexin V-FITC and PI staining. **(B)** The HT29 cells were treated with porric acid E for 48 h, and apoptosis-associated proteins including Bcl-2, Bax and cleaved caspase-3 were examined by Western blot analysis. **(C)** Cell viability of HT29 cells was analyzed using Alamar Blue staining after porric acid E treatment in the presence or absence of chloroquine (CQ). **(D)** Cell viability of HT29 cells was evaluated by Alamar Blue assay after porric acid E exposure with or without Z-VAD-FMK. Data are presented as means ± SEM. *P < 0.05, **P < 0.01.

Since porric acid E treatment was able to induce autophagy in CRC cells, we sought to investigate whether porric acid E-mediated autophagy could contribute to cell death in the form of apoptosis. Cell survival was analyzed by staining with Annexin V-FITC/propidium iodide (PI) staining followed by flow cytometry. The results showed that porric acid E treatment induced early- and late-phase apoptosis in HT29 cells in a dose-dependent manner (Fig. 4A). In addition, a high-dose (80 nM) of porric acid E could induce a nearly 1.7-fold increase in the number of apoptotic cells compared with the low-dose (40 nM) treated group in HT29 cells (Fig. 4A). Western blot analysis also revealed that pro-apoptotic protein cleaved caspase-3 was increased, but antiapoptotic protein Bcl-2 was suppressed by porric acid E treatment (Fig. 4B). To further explore the role of autophagy, HT29 cells were treated with porric acid E in combination with chloroquine (CQ), an inhibitor of autophagy. Importantly, we found that the inhibitory effect of porric acid E on HT29 cells was notably reversed in the presence of CQ (Fig. 4C). Then, to elucidate the particular role of apoptosis in porric acid E mediated cellular suppression, we discovered that a caspase inhibitor Z-VAD-FMK, at least, partly rescued the cell viability in HT29 cells (Fig. 4D). These findings suggested that autophagic cell death is indeed implicated in porric acid E-mediated cytotoxicity.

### Porric acid E treatment exerts antitumor effect of HT29 subcutaneous xenografts *in vivo*

**Figure 5:**
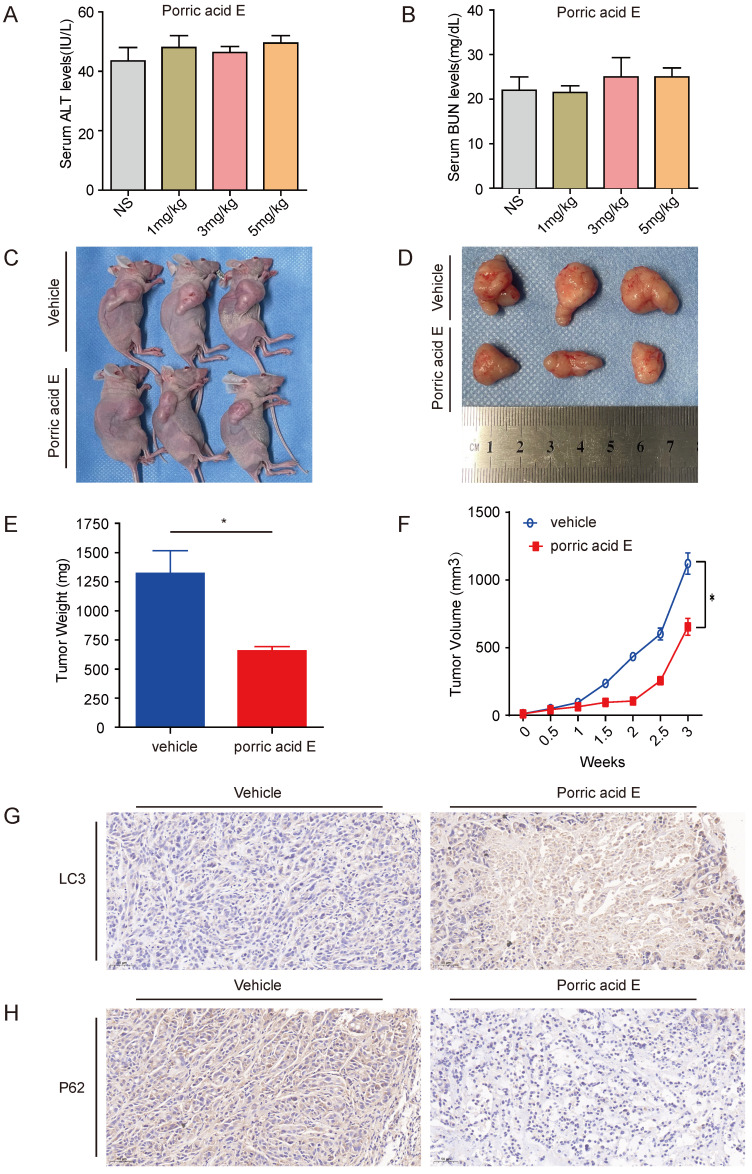
** Porric acid E treatment inhibited tumorigenesis of HT29 subcutaneous xenografts *in vivo*. (A, B)** Nude mice were given IP injections of normal saline solution or escalating doses of porric acid E. Liver (A) and renal (B) functions were examined 72 h after injection by measurement of circulating serum ALT and BUN levels. (C) Images of porric acid E treatment in HT29 tumor-bearing nude mice. **(D)** 21 days post porric acid E treatment, xenograft tumors were photographed. **(E, F)** Tumor weight (E) and volume (F) in HT29 tumor-bearing mice upon treatment with a vehicle of porric acid E. **(G, H)** Representative IHC images of LC3 (G) and p62 (H) in vehicle- or porric acid E-treated tumor tissues.

To examine the therapeutic efficiency of porric acid E *in vivo*, the toxicity profile of porric acid E was examined. Nude mice were given intravenous injections of either saline or porric acid E (1, 3 or 5 mg/kg). Liver and kidney function was assessed 72 h after injection by measuring the circulating alanine aminotransferase (ALT) and blood urea nitrogen (BUN) levels, respectively. Porric acid E doses of up to 5 mg/kg did not affect the liver or kidney function (Fig. 5A, B). Then, a subcutaneous xenograft tumor model of HT29 cells in nude mice was established. Ten days after the CRC cells inoculation, porric acid E at a dose of 3 mg/kg or vehicle control was administrated via tail vein into mice with established tumors. We found that porric acid E could significantly suppress tumor growth in mice 21 days post-treatment, while the vehicle-treated group exhibited stronger tumorigenesis (Fig. 5C, D). Similarly, the tumor weight and tumor volume were notably lower in the porric acid E-treated group than that in the vehicle-treated group (Fig. 5E, F). Additionally, IHC assays demonstrated that porric acid E treatment obviously enhanced the accumulation of LC3 and reduced the expression of p62 in tumor tissues, suggesting that porric acid E could induce autophagy in the nude mouse xenograft model (Fig. 5G, H). Collectively, these results indicated that porric acid E treatment is effective in inhibiting the tumorigenic potential of HT29 cells in a subcutaneous xenograft model, and autophagy is involved in this process.

### Beclin-1 is the potential target protein of porric acid E

**Figure 6:**
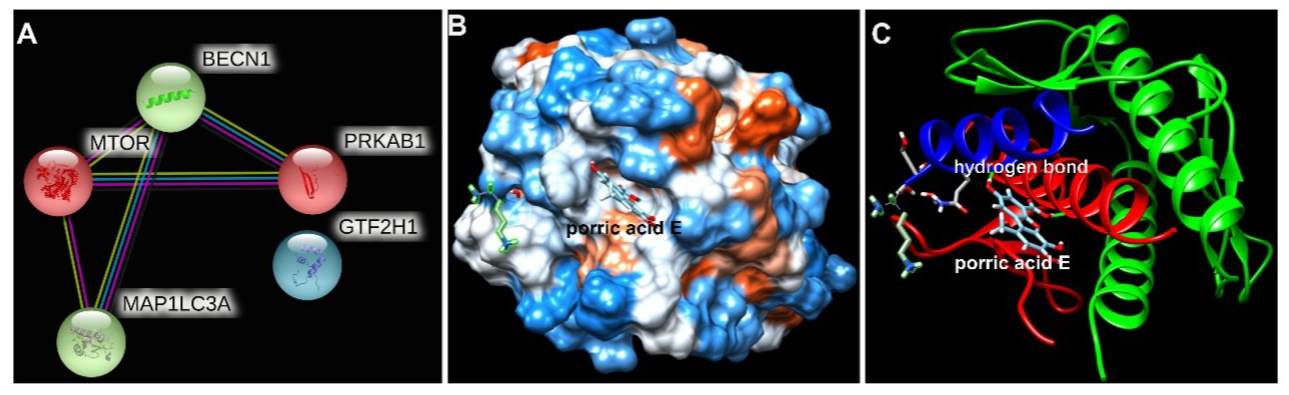
** The interaction between porric acid E and core target. (A)** Protein-protein interactions (PPIs) screened the core target of porric acid E; **(B, C)** molecular docking predicted the interaction between Beclin-1 and porric acid E, and the light blue molecule represents porric acid E.

To further explore the potential interaction of porric acid E and autophagy-associated proteins, an in-silico approach was applied in this study. Protein-protein interaction (PPI) screened the core target of porric acid E was Beclin-1 (BECN1) (Fig. 6A). We found that molecular docking predicted the interaction between Beclin-1 and porric acid E. The results showed that porric acid E was exposed in the active pocket of Beclin-1 and interacted with amino acid residues of Tyr 256 and Trp 438 through hydrogen bond (Fig. 6B, C), with the binding energy of -6.522922 kcal/mol, indicating efficient interaction between Beclin-1 and porric acid E.

## Discussion

Currently, all existing therapeutic methods for CRC have a risk of tumor recurrence 21. Numerous studies have revealed several resistance mechanisms that lead to a reduction in the efficacy of anti-cancer agents on cancer cells 22. Thus, improving the therapeutic outcome of anti-cancer approaches and developing alternative treatments for CRC is an urgent task. In this study, we are the first to isolate a natural molecule, porric acid E, from Rhytidhysteron sp. BZM-9 and evaluate its anticancer effects on CRC both *in vitro* and *in vivo*. We determined that porric acid E exerted its antitumor ability by inhibiting cell viability and inducing apoptosis *in vitro*, and suppressing the growth of HT29-derived xenograft tumors *in vivo*. Moreover, we demonstrated that these effects were dependent on autophagy.

Endophytes are important chemical synthesizers inside plants 23. The different natural compounds produced by endophytic fungi have unique bioactivities and structures. It represents a giant reservoir that affords enormous potential for exploitation in the medical field 24. However, a large number of plant species endophytes have not been evaluated for their anticancer efficacy currently. Merely 0.75-1.50% of known plant species have been explored for their endophytes 25. In recent years, endophytic fungi have attracted much attention due to their advantages such as short culture period, easy preservation, low cost, reproducibility, and the ability to produce active metabolites similar to or the same as those of medicinal plants 26. Attempts have been made to isolate and identify multifarious bioactive metabolites from endophytic fungi 27. The inhibitory activity of ethyl acetate extract derived from Rhytidhysteron sp. BZM-9 on CRC cells aroused our interest. Surprisingly, this current study is the first to confirm that porric acid E, a molecule isolated from Rhytidhysteron sp. BZM-9, exhibits significantly anticancer properties against CRC. Porric acid E belongs to the class of dibenzofuran derivatives, which are typically polyfunctionalized and occur as either totally aromatized or as partially saturated derivatives 28. Based on our findings, we concluded that porric acid E possesses potent inhibitory effects on CRC cell lines by inhibiting cell viability and promoting apoptosis.

Apoptosis and necrosis are the two types of cell death 29, 30. Apoptosis promotion has been proven to be a pivotal underlying mechanism in CRC treatment 31. Apoptosis-associated protein expression levels, such as Bcl-2 and Bax, have been documented as a crucial factor in determining the fate of cancer cells to undergo apoptosis, and the majority of apoptosis occurs in a caspase-3-dependent manner 32. Our data showed that porric acid E can markedly activate the intrinsic apoptotic pathway in CRC cells, as evidenced by the increased protein levels of cleaved caspase-3 and decreased expression of Bcl-2, which were consistent with some of the previous studies 29, 33. Furthermore, flow cytometry assays also revealed that porric acid E treatment notably induced the apoptosis of HT29 cells in a dose-dependent manner. However, a dose of 120 nM porric acid E did not suppress cell viability in NCM460 cells (supplementary Fig. S4), which suggested that porric acid E might be a potential therapeutic agent to induce apoptosis of CRC cells without affecting healthy cells.

Autophagy is a crucial cellular catabolic pathway that regulates cell death, and the interconnections between apoptosis and autophagy are also complicated 34. Depending on cell types, intracellular molecules and extracellular stimuli involved, autophagy might be cytoprotective or cytotoxic 35. Accumulating evidence proposed that autophagy might antagonize apoptosis 36, 37, which indicated that cancer cells could potentiate autophagy to reduce apoptosis. In addition, inhibition of autophagic flux could enhance the pro-apoptotic ability of therapeutic modalities when treating CRC cells 38. Some studies revealed that inhibiting autophagy decreased the apoptosis induced by photodynamic therapy, indicating autophagy could also exhibit a cytotoxic effect 39. In this study, to confirm the role of porric acid E-induced autophagy in CRC cells, HT29 cells were treated with porric acid E in the presence of autophagy inhibitor CQ. The results showed that the inhibition of autophagy by CQ could alleviate the porric acid E-mediated anticancer efficiency in CRC cells, as evidenced by increased cell viability. Our data revealed that autophagy may present a potent cytotoxic effect on porric acid E-treated CRC cells.

The bioactive metabolites generated from endophytic fungi belong to different structural groups such as quinones, terpenoids, steroids, coumarin, phenols, etc. 40. Rhytidhysteron sp. BZM-9 is an endophyte isolated from the leaves of Leptospermum brachyandrum. Our previous studies showed that we successfully extracted some types of compounds from Rhytidhysteron sp. BZM-9, including phthalide derivatives 41, isocoumarin derivatives 42 and chlorinated cyclopentene derivatives 43. Furthermore, most of these derivatives exhibited cytotoxic activities against human CRC cell lines or human hepatoma cell lines, but none of them were employed on subcutaneous xenograft models due to limited water solubility. In this study, we isolated a highly water-soluble natural molecule porric acid E from Rhytidhysteron sp. BZM-9, and investigated the anticancer effect of porric acid E *in vivo* for the first time. We verified that porric acid E significantly suppressed the growth rate of subcutaneous tumors in nude mice. Moreover, LC3 and p62 protein levels were also changed in porric acid E-treated tumor tissues. These results further revealed that the autophagy pathway was involved in porric acid E-mediated inhibition of CRC *in vivo*. Molecular docking indicated the docking sites Tyr 256 and Trp 438 of Beclin-1 might be a vital role in the recognition and docking of porric acid E, which still needs further study. Meanwhile, organ damage evaluated by biochemical analysis (ALT and BUN levels) demonstrated that Porric acid E has no significant toxic effects *in vivo*. This suggests that porric acid E might work as a promising candidate agent against CRC.

In summary, we isolated a natural compound porric acid E from Rhytidhysteron sp. BZM-9 for developing an anti-cancer drug for CRC treatment. Our study revealed that porric acid E potently inhibited the growth of CRC *in vitro* and *in vivo*. This anticancer effect is mediated by suppressing proliferation/cell viability, promoting apoptosis, and inducing autophagy. Moreover, inhibition of autophagy could remarkably abrogate porric acid E-mediated anticancer efficiency in CRC cell lines. Altogether, we provided evidence for the therapeutic application of porric acid E for CRC treatment. However, further clinical trials are needed to determine the efficacy and safety of porric acid E.

## Supplementary Material

HRESIMS and NMR spectra of porric acid E are available as Supplementary Information (Supplementary Fig. S1-S3).

## Figures and Tables

**Table 1:** ^1^H (600 MHz) and ^13^C (150 MHz) NMR spectral data of porric acid E in CD_3_OD

Position	*δ*_H_ (*J* in Hz)	*δ*_C_	Types of C
1		126.9	C
2	6.68, s	116.3	CH
3		146.3	C
4		130.9	C
4a		141.3	C
5a		164.8	C
6		97.5	C
7	6.35, d, *J* = 1.8 Hz	100.6	CH
8		165.0	C
9	7.23, d, *J* = 1.8 Hz	104.2	CH
9a		139.1	C
9b		109.8	C
10	2.66, s	23.9	CH_3_
11		165.4	C

## Abbreviations

CRC: colorectal cancer
5-FU: fluorouracil
TEM: transmission electron microscopy
CQ: chloroquine
ALT: alanine aminotransferase
BUN: blood urea nitrogen
PPI: protein-protein interaction
IHC: Immunohistochemistry

## References

[1] Dekker E, Tanis PJ, Vleugels JLA, Kasi PM, Wallace MB (2019). Colorectal cancer. Lancet (London, England).

[2] Schreuders EH, Ruco A, Rabeneck L, Schoen RE, Sung JJ, Young GP (2015). Colorectal cancer screening: a global overview of existing programmes. Gut.

[3] Simon K (2016). Colorectal cancer development and advances in screening. Clinical interventions in aging.

[4] Miller KD, Nogueira L, Mariotto AB, Rowland JH, Yabroff KR, Alfano CM (2019). Cancer treatment and survivorship statistics, 2019. CA: a cancer journal for clinicians.

[5] Fabiani R (2020). Antitumoral Properties of Natural Products. Molecules (Basel, Switzerland).

[6] Rayan A, Raiyn J, Falah M (2017). Nature is the best source of anticancer drugs: Indexing natural products for their anticancer bioactivity. PLoS ONE.

[7] Franco YEM, Okubo MY, Torre AD, Paiva PP, Rosa MN, Silva VAO (2019). Coronarin D Induces Apoptotic Cell Death and Cell Cycle Arrest in Human Glioblastoma Cell Line. Molecules (Basel, Switzerland).

[8] Gomes INF, Silva-Oliveira RJ, Oliveira Silva VA, Rosa MN, Vital PS, Barbosa MCS (2019). Annona coriacea Mart. Fractions Promote Cell Cycle Arrest and Inhibit Autophagic Flux in Human Cervical Cancer Cell Lines. Molecules (Basel, Switzerland).

[9] El-Bondkly EAM, El-Bondkly AAM, El-Bondkly AAM (2021). Marine endophytic fungal metabolites: A whole new world of pharmaceutical therapy exploration. Heliyon.

[10] Stierle A, Strobel G, Stierle D (1993). Taxol and taxane production by Taxomyces andreanae, an endophytic fungus of Pacific yew. Science (New York, NY).

[11] Hridoy M, Gorapi MZH, Noor S, Chowdhury NS, Rahman MM, Muscari I (2022). Putative anticancer compounds from plant-derived endophytic fungi: A review. Molecules.

[12] Zhang S, Wang W, Tan J, Kang F, Chen D, Xu K (2021). Rhytidhyesters A - D, 4 new chlorinated cyclopentene derivatives from the endophytic fungus *Rhytidhysteron* sp. BZM-9. Planta Med.

[13] Boya P, Reggiori F, Codogno P (2013). Emerging regulation and functions of autophagy. Nature cell biology.

[14] Domagala A, Stachura J, Gabrysiak M, Muchowicz A, Zagozdzon R, Golab J (2018). Inhibition of autophagy sensitizes cancer cells to Photofrin-based photodynamic therapy. BMC Cancer.

[15] Xiaokaiti Y, Li X (2020). Natural Product Regulates Autophagy in Cancer. Advances in experimental medicine and biology.

[16] Tian Y, Lin X, Zhou X, Liu Y (2018). Phenol derivatives from the sponge-derived fungus Didymellaceae sp. SCSIO F46. Frontiers in chemistry.

[17] Diasio RB, Harris BE (1989). Clinical pharmacology of 5-fluorouracil. Clinical pharmacokinetics.

[18] Tseng HC, Liu WS, Tyan YS, Chiang HC, Kuo WH, Chou FP (2011). Sensitizing effect of 3-methyladenine on radiation-induced cytotoxicity in radio-resistant HepG2 cells *in vitro* and in tumor xenografts. Chemico-biological interactions.

[19] Eimer S, Belaud-Rotureau MA, Airiau K, Jeanneteau M, Laharanne E, Véron N (2011). Autophagy inhibition cooperates with erlotinib to induce glioblastoma cell death. Cancer biology & therapy.

[20] Katayama M, Kawaguchi T, Berger MS, Pieper RO (2007). DNA damaging agent-induced autophagy produces a cytoprotective adenosine triphosphate surge in malignant glioma cells. Cell death and differentiation.

[21] Johdi NA, Sukor NF (2020). Colorectal cancer immunotherapy: options and strategies. Front Immunol.

[22] Tacconi C, Ungaro F, Correale C, Arena V, Massimino L, Detmar M (2019). Activation of the VEGFC/VEGFR3 Pathway Induces Tumor Immune Escape in Colorectal Cancer. Cancer research.

[23] Chandra S (2012). Endophytic fungi: novel sources of anticancer lead molecules. Applied microbiology and biotechnology.

[24] Gunatilaka AA (2006). Natural products from plant-associated microorganisms: distribution, structural diversity, bioactivity, and implications of their occurrence. Journal of natural products.

[25] Singh A, Singh DK, Kharwar RN, White JF, Gond SK (2021). Fungal endophytes as efficient sources of plant-derived bioactive compounds and their prospective applications in natural product drug discovery: Insights, avenues, and challenges. Microorganisms.

[26] Li SJ, Zhang X, Wang XH, Zhao CQ (2018). Novel natural compounds from endophytic fungi with anticancer activity. Eur J Med Chem.

[27] Chandra S, Bandopadhyay R, Kumar V, Chandra R (2010). Acclimatization of tissue cultured plantlets: from laboratory to land. Biotechnology letters.

[28] Millot M, Dieu A, Tomasi S (2016). Dibenzofurans and derivatives from lichens and ascomycetes. Nat Prod Rep.

[29] Yan S, Tang D, Hong Z, Wang J, Yao H, Lu L (2021). CD133 peptide-conjugated pyropheophorbide-a as a novel photosensitizer for targeted photodynamic therapy in colorectal cancer stem cells. Biomaterials science.

[30] Tang D, Fu G, Li W, Sun P, Loughran PA, Deng M (2021). Maresin 1 protects the liver against ischemia/reperfusion injury via the ALXR/Akt signaling pathway. Molecular medicine (Cambridge, Mass).

[31] Wei MF, Chen MW, Chen KC, Lou PJ, Lin SY, Hung SC (2014). Autophagy promotes resistance to photodynamic therapy-induced apoptosis selectively in colorectal cancer stem-like cells. Autophagy.

[32] Yoo ES, Choo GS, Kim SH, Woo JS, Kim HJ, Park YS (2019). Antitumor and apoptosis-inducing effects of piperine on human melanoma cells. Anticancer Res.

[33] Lu P, Xu M, Xiong Z, Zhou F, Wang L (2019). Fusobacterium nucleatum prevents apoptosis in colorectal cancer cells via the ANO1 pathway. Cancer management and research.

[34] Huang Q, Ou YS, Tao Y, Yin H, Tu PH (2016). Apoptosis and autophagy induced by pyropheophorbide-α methyl ester-mediated photodynamic therapy in human osteosarcoma MG-63 cells. Apoptosis: an international journal on programmed cell death.

[35] Ning ST, Lee SY, Wei MF, Peng CL, Lin SY, Tsai MH (2016). Targeting Colorectal Cancer Stem-Like Cells with Anti-CD133 Antibody-Conjugated SN-38 Nanoparticles. ACS applied materials & interfaces.

[36] Shimizu S, Takehara T, Hikita H, Kodama T, Tsunematsu H, Miyagi T (2012). Inhibition of autophagy potentiates the antitumor effect of the multikinase inhibitor sorafenib in hepatocellular carcinoma. International journal of cancer.

[37] Song J, Guo X, Xie X, Zhao X, Li D, Deng W (2011). Autophagy in hypoxia protects cancer cells against apoptosis induced by nutrient deprivation through a Beclin1-dependent way in hepatocellular carcinoma. Journal of cellular biochemistry.

[38] Yue W, Hamaï A, Tonelli G, Bauvy C, Nicolas V, Tharinger H (2013). Inhibition of the autophagic flux by salinomycin in breast cancer stem-like/progenitor cells interferes with their maintenance. Autophagy.

[39] Lange C, Lehmann C, Mahler M, Bednarski PJ (2019). Comparison of Cellular Death Pathways after mTHPC-mediated Photodynamic Therapy (PDT) in Five Human Cancer Cell Lines. Cancers.

[40] Kaul S, Gupta S, Ahmed M, Dhar MK (2012). Endophytic fungi from medicinal plants: A treasure hunt for bioactive metabolites. Phytochemistry Reviews.

[41] Zhang S, Chen D, Kuang M, Peng W, Chen Y, Tan J (2021). Rhytidhylides A and B, Two New Phthalide Derivatives from the Endophytic Fungus Rhytidhysteron sp. BZM-9. Molecules (Basel, Switzerland).

[42] Zhang S, Kang F, Tan J-B, Chen D, Kuang M, Wang W-X (2021). (±)-Rhytidhymarins A and B, two pairs of new isocoumarin derivatives from endophytic fungus Rhytidhysteron sp. BZM-9.

[43] Zhang S, Wang W, Tan J, Kang F, Chen D, Xu K (2021). Rhytidhyesters A - D, 4 New Chlorinated Cyclopentene Derivatives from the Endophytic Fungus Rhytidhysteron sp. BZM-9. Planta medica.

