# Supplementary material for: Porric acid E, a natural compound from Rhytidhysteron sp. BZM-9, suppresses colorectal cancer growth via an autophagy-dependent pathway
Source: J Cancer. 2022 Oct 31;13(14):3554–65. doi: 10.7150/jca.77588 (PMC9723991; doi:10.7150/jca.77588)

Porric acid E, a natural compound from Rhytidhysteron sp. BZM-9, suppresses colorectal cancer growth via an autophagy-dependent pathway

Supplementary Information

Figure S1 HRESIMS spectrum of porric acid E.

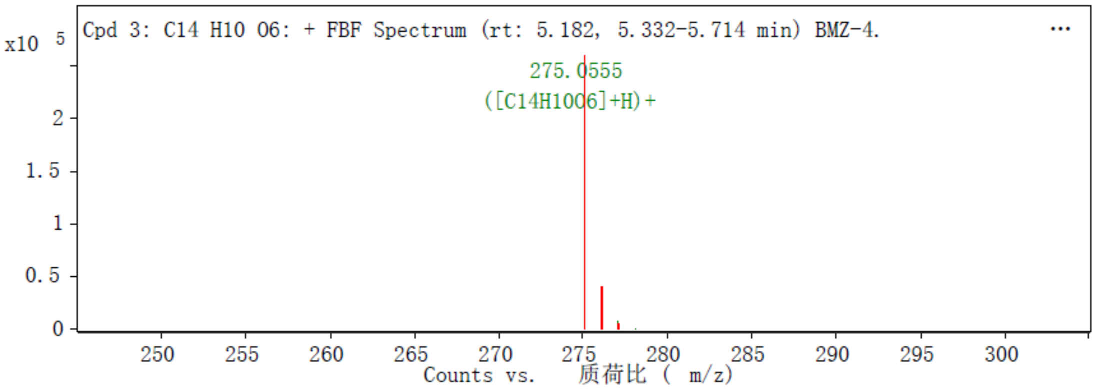

Figure S2 ^{1}H NMR spectrum of porric acid E.

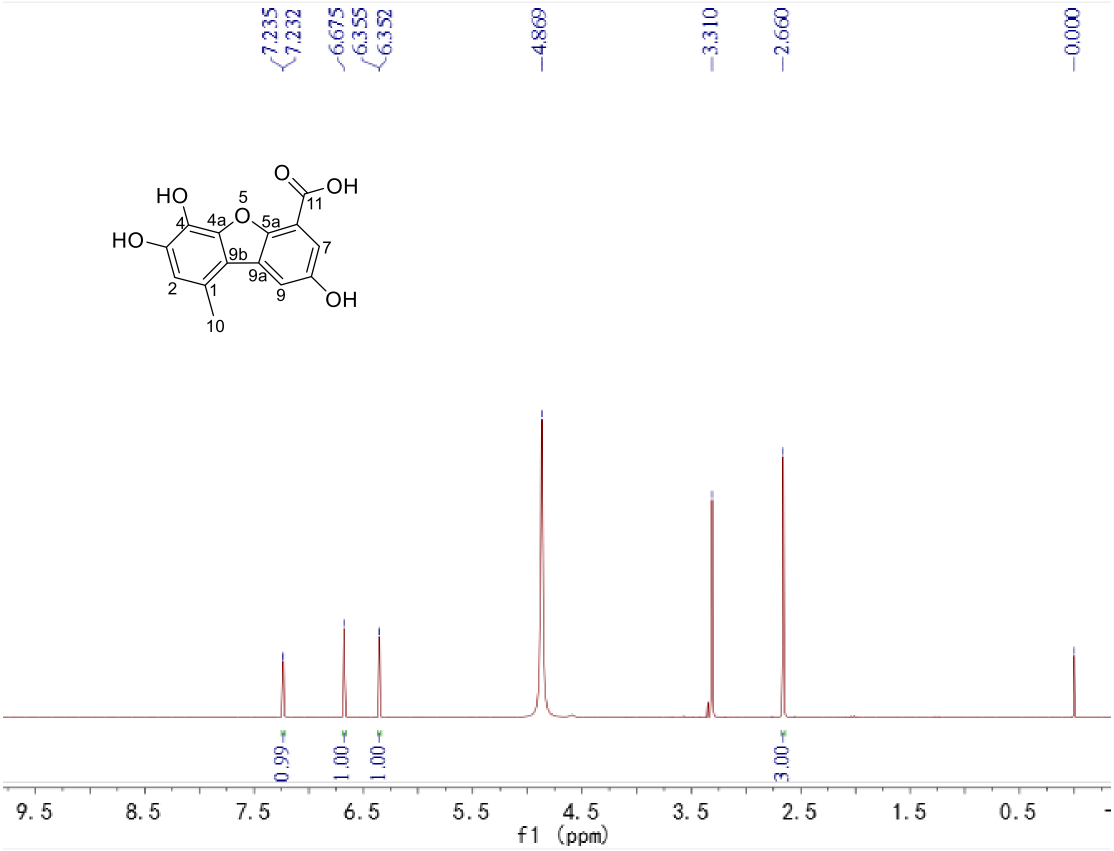

Figure S3 ^{13}C NMR spectrum of porric acid E

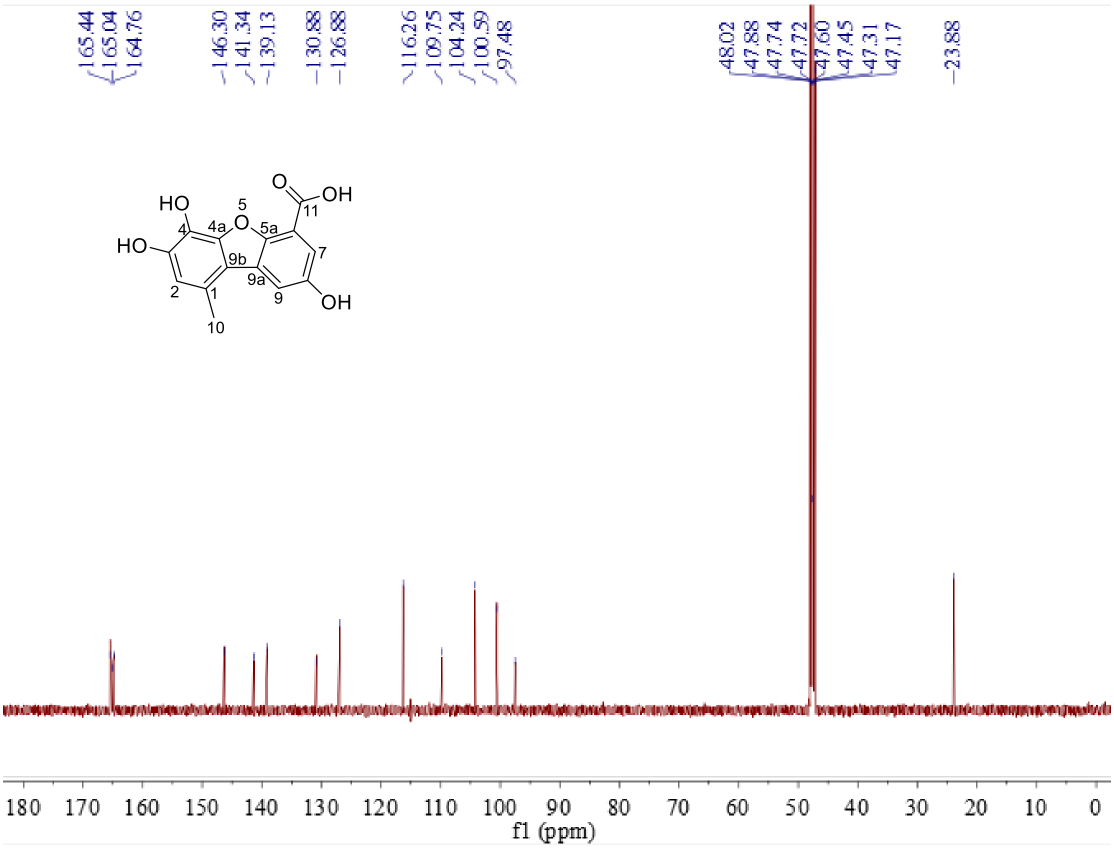

Figure S4: Porric acid E preferentially inhibits the proliferation of different cancer cells as compared with the normal human colonic mucosal epithelial cells. Liver cancer cell line (HepG2), nasopharyngeal carcinoma cell line (5-8F), triple-negative breast cancer cell line (MDA-MB-231) and normal human colon mucosal epithelial cell line (NCM460) were treated with different concentrations of porric acid E for 48 h and subjected to Alamar Blue cell viability examination.

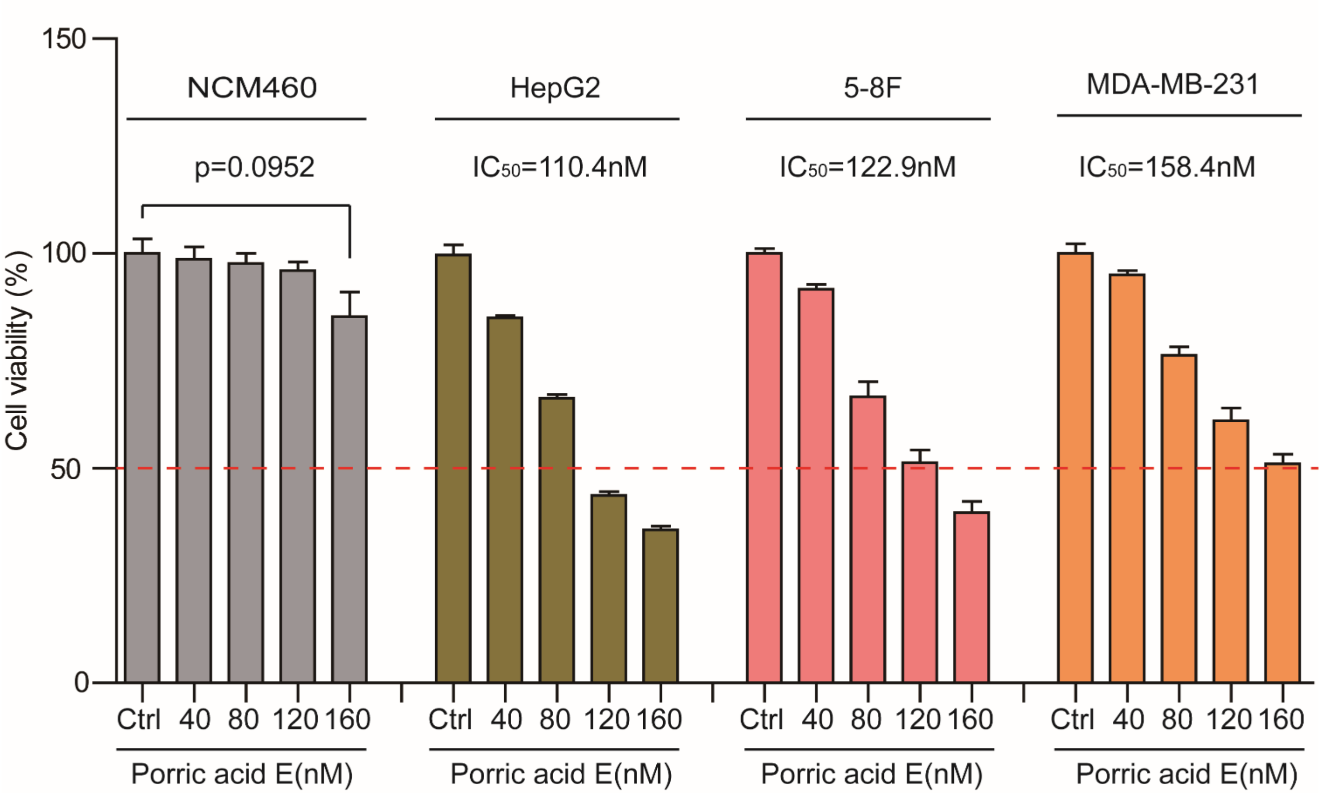

Supplement: Supplementary file 1 — HRESIMS and NMR spectra of porric acid E are available as Supplementary Information (Supplementary Fig. S1-S3). [file jcav13p3554s1.pdf]
